# Patient-Caregiver Portal System in Palliative Oncology: Assessment of Usability and Perceived Benefit

**DOI:** 10.2196/47624

**Published:** 2023-11-02

**Authors:** Margaret L Longacre, Marcin Chwistek, Cynthia Keleher, Mark Siemon, Brian L Egleston, Molly Collins, Carolyn Y Fang

**Affiliations:** 1 College of Health Sciences Arcadia University Glenside, PA United States; 2 Supportive Oncology and Palliative Care Program Fox Chase Cancer Center Philadelphia, PA United States; 3 Web Technologies Department Fox Chase Cancer Center Philadelphia, PA United States; 4 Biostatistics and Bioinformatics Facility Fox Chase Cancer Center Philadelphia, PA United States; 5 Cancer Prevention and Control Program Fox Chase Cancer Center Philadelphia, PA United States

**Keywords:** caregiving, patient portal, health policy, palliative oncology, oncology, engagement, family caregiver, caregiver, communication, usage, usability, clinical care, cancer

## Abstract

**Background:**

The engagement of family caregivers in oncology is not universal or systematic.

**Objective:**

We implemented a process intervention (ie, patient-caregiver portal system) with an existing patient portal system to (1) allow a patient to specify their caregiver and communication preferences with that caregiver, (2) connect the caregiver to a unique caregiver-specific portal page to indicate their needs, and (3) provide an electronic notification of the dyad’s responses to the care team to inform clinicians and connect the caregiver to resources as needed.

**Methods:**

We assessed usability and satisfaction with this patient-caregiver portal system among patients with cancer receiving palliative care, their caregivers, and clinicians.

**Results:**

Of 31 consented patient-caregiver dyads, 20 patients and 19 caregivers logged in. Further, 60% (n=12) of patients indicated a preference to communicate equally or together with their caregiver. Caregivers reported high emotional (n=9, 47.3%), financial (n=6, 31.6%), and physical (n=6, 31.6%) caregiving-related strain. The care team received all patient-caregiver responses electronically. Most patients (86.6%, 13/15 who completed the user experience interview) and caregivers (94%, 16/17 who completed the user experience interview) were satisfied with the system, while, of the 6 participating clinicians, 66.7% agreed “quite a bit” (n=1, 16.7%) or “very much” (n=3, 50%) that the system allowed them to provide better care.

**Conclusions:**

Our findings demonstrate system usability, including a systematic way to identify caregiver needs and share with the care team in a way that is acceptable to patients and caregivers and perceived by clinicians to benefit clinical care. Integration of a patient-caregiver portal system may be an effective approach for systematically engaging caregivers. These findings highlight the need for additional research among caregivers of patients with less advanced cancer or with different illnesses.

## Introduction

### Caregiving in Cancer

According to a 2020 national survey by the National Alliance for Caregiving (NAC) and the American Association of Retired Persons, approximately 19.2% of the US population, or 47.9 million individuals, provided informal care to an adult in 2019 [1]. Of these caregivers, slightly over 2.8 million (or 6%) provided care due to cancer as the primary reason. It is likely that more caregivers were supporting someone with cancer given that many of the care recipients in the survey indicated comorbidities (45%) [1]. Caregiving in cancer can involve a high number of hours and varied, demanding tasks, such as monitoring symptoms, communicating with health care professionals, and performing nursing tasks [1,2].

Caregivers are shown to experience high levels of caregiving-related financial, physical, and emotional strain [1], and in the cancer context, caregiving is particularly challenging due to emotional strain [3,4]. Experiencing elevated stress and poorer emotional health as a caregiver can have adverse implications on patients due to potential congruence between a caregiver and patient’s level of distress [5,6]. It is also possible that there might be higher system spending and poorer quality ratings when a patient as well as caregiver’s needs go unmet or when experiencing distress [7].

### Identifying and Engaging Caregivers in Care

Though many oncology-specific caregiving interventions have been developed in recent years [8,9], few align with practice recommendations and policies seeking to systematically integrate caregivers into care [10]. Specifically, as early as 2001, recommendations emerged to integrate caregivers in older adult care to improve patient outcomes [11,12]. Calls to integrate caregivers in all aspects of care are increasingly evident in oncology [10,13], while the National Academies of Medicine and the American Institutes for Research (AIR) notes the priority of enhancing the policy and practice “infrastructure” to deliver patient- and family-centered care [14,15]. State laws in many states also now require the identification of a caregiver (if available) in a patient’s electronic medical record, that the caregiver be informed when the patient is transferred, and that the caregiver receive training (broadly defined) when the patient is discharged (ie, the Caregiver Advise, Record, and Enable Act) [16]. Proponents of patient- and family-centered care models suggest that better engagement of patients and their caregivers will result in improved patient safety and care quality, better patient experiences and satisfaction, lower costs, and higher clinician satisfaction [14,17]. There are important considerations, however, when engaging caregivers in care, including preserving patient autonomy as, according to Clayman’s Autonomy framework [18] and related work [19] caregivers can be “autonomy enhancing” or “autonomy detracting.”

### Embracing Systems and Technology to Identify, Engage, and Connect Caregivers in Care

Today, caregivers are not systematically identified or engaged in care, their needs are often unrecognized and unmet, and they experience elevated stress and psychological health deficits. For example, an assessment of the implementation of the Caregiver Advise, Record, and Enable Act in a large health system in Pennsylvania noted the inclusion of the caregiver in the electronic health record, but did not include notation of caregiver notification about patient discharge or education or training, suggesting a missed opportunity to fully benefit caregivers and patients [20]. The AIR’s Roadmap for Patient and Family Engagement in Healthcare Practice and Research [14] suggests the need to promote patient and family-centered care models and to explore the use of existing technology—for example, patient portal systems—in doing so. These suggestions align with trends in use of patient portals showing gradual increases over the past several years, with nearly 40% of US adults reporting they had engaged with their portal at least once in the previous 12 months [21]. Importantly, findings also suggest that a care team’s recommendations to use the portal increases the likelihood of engagement, which suggests a systems-based approach might be beneficial [22]. Similarly, a recent scoping review of portal use by caregivers demonstrated that caregivers, when engaged as a registered user, see greater benefit with use compared to being a nonregistered user [23]. Together, these findings highlight the potential to systematically engage caregivers via patient portals.

### Purpose

We previously developed [24,25] a patient-portal based process intervention, entitled patient-caregiver portal system, in accordance with concepts from the *Patient and Family Engaged Care* Framework [17], the Roadmap for Patient and Family Engagement in Healthcare Practice and Research [14], and Clayman’s Autonomy framework [18] along with related work [26,27]. The patient-caregiver portal system is designed to be embedded within the health care institution’s patient portal system and (1) allow a patient to specify their primary caregiver and their communication preferences with that caregiver in the health care setting, (2) connect the caregiver to a unique portal page to indicate their needs as a caregiver, and (3) provide an electronic notification of the dyad’s responses to the care team to inform clinicians and connect the caregiver to resources as needed. The purpose of this pilot study was to assess use and perception of benefit of the patient-caregiver portal system among patients, caregivers, and clinicians in an outpatient palliative oncology setting.

## Methods

### Participants

Participants in this study included cancer patients receiving palliative care, their caregivers, and their palliative care oncology clinicians. Eligible patients (1) were 18 years of age or older, (2) receiving outpatient cancer care at consent, (3) referred to palliative care, (4) had a caregiver 18 years of age or older involved in care (on-site not required), (5) were able to read or communicate in English, and (6) had internet capability or ability to access the portal system if using the system away from the cancer center. Eligible caregivers were (1) 18 years of age or older, (2) providing informal care to the study-eligible patient, (3) able to read or communicate in English, and (4) with internet capability or ability to access the portal system if using it away from the cancer center. Eligible clinicians included those providing palliative care services and involved in the care of the patient-caregiver dyads participating in the study.

### Ethical Considerations

This study was approved by the Cancer Center’s institutional review board (#18-8005) and all participants—patients, caregivers, and clinicians—provided informed consent. Participation was voluntary and participants were informed that they could choose not to answer a question or stop participating at any time.

### Participant Recruitment

Participant recruitment was initiated in February 2020, but was briefly paused in March 2020 for patient safety due to the COVID-19 pandemic. Recruitment was restarted in May 2020 and continued until October 2021 (with the majority of recruitment occurring between May 2020-July 2021). Due to COVID-19, most patients had their appointments converted to telehealth visits. The study research assistant (RA) introduced the study to a patient and caregiver virtually via telephone. If the patient and caregiver were interested in participating, the study RA reviewed the study informed consent document and secured informed consent from the patient and caregiver individually.

### Study Procedures

Once the enrolled patient logged in to their patient portal system, they were prompted to answer questions about their preferred primary caregiver, including that caregiver’s contact information, and their preferred communication with the caregiver in health care. Next, an invitation was then sent to the caregiver to login to the portal system using a unique username and password. Once the caregiver logged in, this caregiver received an invitation to complete the caregiver-specific questions about their preparation to be a caregiver; caregiving-related emotional, physical, and financial strain [3,28]; and need for information about addressing emotional, physical, and financial strain, communicating with the patient’s care team, and about managing patient symptoms. The selection of these questions were based on focus group input and prior literature recommending that clinic-based assessments be concise, related to constructs of quality of life, and actionable, and have been validated in assessing physical, emotional, and financial caregiving-related strain and overall caregiving-related strain [13,28,29].

Upon completion of the caregiver questions, the patient and caregiver responses were sent electronically to the care team both through the portal system and through a HIPAA (Health Insurance Portability and Accountability Act)-compliant email. Moreover, to assist in responsiveness to caregiver needs, the Department of Social Work was alerted if a caregiver reported heightened strain (responses of 3 or above on a 1-5 Likert scale) in any of the 3 caregiving-related strain domains (physical, emotional, or financial). The patients and caregivers were invited to complete a user experience interview once they completed use of the patient-caregiver portal system and had at least 1 follow-up appointment with their primary palliative care clinician. After the follow-up appointment, clinicians were asked to complete a survey on the perceived benefit of the system for clinical care delivery and their satisfaction with this process.

### Measurement

#### Overview

We collected the following information from patients, caregivers, and clinicians.

#### Patient and Caregiver Characteristics

Patient information including age, gender, race, ethnicity and cancer characteristics (eg, date of diagnosis, cancer type, and cancer stage) was abstracted by study staff via a review of medical records. Caregivers self-report demographic information (ie, age, gender, race, ethnicity, education, and household income) using the caregiver survey in the patient-caregiver portal system.

#### System Use by Patients

We collected the following patient use information: (1) system log-in; (2) submission of caregiver information (ie, caregiver’s name, email, telephone, address, and the caregiver’s relationship to the patient); and (3) completion of the communication preference item (ie, “How do you prefer to communicate with your doctor or care team when/if this caregiver is involved?”). Response options included: I usually prefer to communicate by myself or independently; I usually prefer to communicate together or equally with my caregiver; or I usually prefer that my caregiver communicate for me.

#### System Use by Caregivers

Caregiver use information included (1) system login following the email invitation and (2) completion of the caregiver-specific questions. Caregivers’ perceived preparation was assessed using the following question: How prepared do you feel to assist the patient (not at all, a little bit, somewhat, quite a bit, and very much). For caregiver strain, caregivers were asked about their level of (physical or emotional or financial) strain: How [emotionally stressful/physical straining/financially straining] would you say that caring for your relative/friend with cancer is for you? (1: not at all to 5: very much) [28]. Finally, caregivers were asked “Which of the following topics do you feel you need more information about?...Managing my physical stress/Managing my emotional stress/Managing my physical stress/Managing the patient’s symptoms/Communicating with the patient’s doctor or care team.” Caregivers selected “yes” or “no” for each topic.

#### Receipt of Information by Care Team

We tracked receipt of patient and caregiver portal responses by the care team through acknowledgment from the clinician (yes or no) as well as referral (yes or no) to social work in cases of caregiver elevated strain on either the emotional, physical, or financial strain items (ie, levels of 3 or higher on a Likert scale of 1: not at all to 5: very much).

#### Patient and Caregiver User Experience

To understand satisfaction with the system, we conducted a brief post–user experience interview by telephone with patients and caregivers, including asking: “overall, were you satisfied with this method to involve a caregiver in care? Why or why not?” The study RA conducted the interviews and captured their responses in an electronic format.

#### Clinician Perception of Benefit

The participating palliative care clinicians completed a survey to assess the perceived benefits of the system and their satisfaction with this process. The survey contained 11 questions that were adapted from the AIR’s Roadmap outcomes [14] regarding the perceived benefit of elements of the system with closed-ended responses ranging from “not at all” to “very much.” Further, two open-ended questions were also included to identify facilitators and barriers to this process: (1) what was most helpful for your practice with this method? and (2) what was most difficult for your practice with this method?

### Analytic Plan

Given the primary goal of this pilot usability study, we conducted descriptive analyses, including percentages and means, to characterize the sample in terms of demographic characteristics, login characteristics, response to stakeholder-specific questions, and clinician survey response pertaining to benefit and satisfaction. Prior to the study, we declared that the system would be deemed feasible for patients if a majority (50% or more of those enrolled) would (1) log-in, (2) report caregiver information, and (3) complete the preference items. Similarly, we declared the system feasible for caregivers if 50% or more of those enrolled would (1) log-in and (2) complete the caregiver items. This benchmark of 50% was informed by related studies of patient portal use [30]. Satisfaction per the user experience interviews for patients and caregivers was determined using an “Integrated Approach” [31] for qualitative analysis. This means beginning with broad or predetermined codes and then allowing subcodes to develop within these broader codes as common to grounded theory. This Yale-developed qualitative method for analysis is effective and efficient when seeking a defined purpose [31]. The patient and caregiver user experience questions about satisfaction were coded as “yes,” “no,” or “unsure” for indication of satisfaction, while responses were listed and synthesized according to related categories for reporting.

## Results

### Overview

In total, 31 patients provided written consent and 20 (64.5%) logged into the portal. Patients who logged in were 62 (median 64, range 35-80) years of age on average, female (n=11, 55%) non-Hispanic White (n=19, 95%), and had late-stage cancer (n=14, 70% stage IV). The patient sample included varied cancer diagnoses, with cancer of the kidney (n=4, 20%), lung (n=4, 20%), and breast (n=3, 15%) being most common with 10% (n=2) as “other” and 5% (n=1) each for endometrial, leukemia, lymphoma, melanoma, multiple myeloma, ovarian, pancreatic, and thyroid cancers. The patients (n=11) who did not log-in were 56 (median 57, range 32-75) years of age on average, 54.5% (n=6) female, predominantly non-Hispanic White (n=8, 72.7%; n=2, 18.2% were Black and n=1, 9% indicated other), and had varied forms of cancer (n=2, 18.2% breast, n=2, 18.2% colon, n=1, 9% for each of the following: bladder, ovarian, pancreatic, prostate, kidney, and Hodgkin lymphoma), and most with stage 4 cancer (n=8, 72.7%). The caregivers who logged in (n=19) were 61 (median 63, range 31-80) years old on average, most often the patient’s spouse (n=14, 73.7%), non-Hispanic White (n=18, 94.7%), female (n=10, 52.6%), had an education level lower than a college degree (n=10, 52.6%), and were working full (n=10, 52.6%; n=2, 10.5% part-time; and n=5, 26.3% retired).

### System Use and Function

Of the 20 patients who logged in to the system, 19 of their caregivers also logged in. All patients and most of the caregivers (n=19, 95%) who logged in to the system answered each of their respective questions. Most patients (n=12, 60%) indicated that they prefer to communicate together or equally with their caregiver when communicating with the care team, followed by communicating independently (n=5, 25%) or delegating communication to the caregiver (n=3, 15%).

Most of the caregivers (14/19, 73.6%) indicated feeling prepared (quite a bit: 47.3% or very much: 26.3%) to assist the patient, while fewer reported feeling “somewhat” (n=4, 21%) or “a little bit” (n=1, 5%) prepared and none felt unprepared. Nearly half (n=9, 47.3%) of the caregivers expressed high (ie, levels 4 and 5) emotional stress, while a lower proportion reported high physical strain (n=6, 31.6% ) and financial strain (n=6, 31.6%). See Table 1 for full responses to the caregiving-related strain questions. Caregivers indicated wanting information about managing their emotional (n=11, 57.8%), financial (n=7, 36.8%), and physical caregiving-related strain (n=2, 10.5%) and information about managing the patient’s symptoms (n=8, 42%) and how to communicate with care teams (n=6, 31.5%). The clinicians received all patient and caregiver responses, and referrals to the Social Work Department were made for all caregivers who reported high strain (as defined above).

**Table 1:** Caregiver responses to patient-caregiver portal system questions (n=19).

Question	1 (not at all), n (%)	2, n (%)	3, n (%)	4, n (%)	5 (very much), n (%)
How emotionally stressful would you say that caring for your relative/friend with cancer is for you?	1 (5.3)	4 (21.1)	5 (26.3)	3 (15.7)	6 (31.6)
How physical straining would you say that caring for your relative/friend with cancer is for you?	5 (26.3)	5 (26.3)	3 (15.8)	5 (26.3)	1 (5.3)
How financially straining would you say that caring for your relative/friend with cancer is for you?	5 (26.3)	6 (31.6)	2 (10.5)	3 (15.8)	3 (15.8)

### User Experience

Patients and caregivers’ satisfaction with the patient-caregiver portal system. Of the 20 patients, 15 were able to complete the user experience interviews. Lack of participation was due to death of the patient (n=3) or their high symptom burden (n=2). Of the patients who completed the user experience interview, 13 (86.6%) were satisfied with the system. Reasons for being satisfied pertained to (1) ease of use, (2) benefit of caregiver integration (ie, when patient cannot interact with the care team or for emergencies), and (3) that the system used current technology. One of these patients also noted the desire to be informed when the care team received the responses, while another noted that communication with the care team was already strong. Of the 15 patients who completed the user experience interviews, 2 patients were not satisfied because of uncertainty that the system was helpful for them, but one of these patients did note that they could see how it could help others.

In total, 17 caregivers completed the user experience interview with 16 caregivers indicating that they were satisfied with the system overall. Reasons for being satisfied included (1) sense of collaboration between patients, caregivers, and care team; (2) simplified interactions; (3) supporting and informing caregivers; and (4) effective strategy compared to telephone. Further, 3 caregivers recommended improvements despite finding the system satisfactory, including having the system be more interactive (eg, live chat) and more tailored to the caregiver in response. In total, 1 caregiver was unsure about being satisfied, but thought it would be better for someone who was caring for a patient more recently diagnosed and early in the care trajectory.

### Clinicians Perception of Benefit

In total, 6 palliative care clinicians (including doctors, nurses, and social workers) who were involved in managing care of the participating dyads completed the clinician user experience survey. Table 2 presents the responses of clinicians with respect to the perceived benefit and helpfulness of the system and impact on care. Open-ended responses identified the following helpful features: (1) it enabled the identification of caregivers, (2) created awareness of caregiver distress and needs, and (3) recognized the need for heightened social work support to assist caregivers. In contrast, the aspects that they found most difficult for their practice included (1) lack of direct integration with Epic electronic medical record, (2) some uncertainty when responses from patients and caregivers would be completed, and (3) some patients’ hesitancy with technology.

**Table 2:** Clinician user experience survey responses (n=6).

Question	Not at all, n (%)	A little bit, n (%)	Somewhat, n (%)	Quite a bit, n (%)	Very much, n (%)
There is benefit in having a method to involve and support caregivers in cancer care.	0 (0)	0 (0)	0 (0)	1 (16.7)	5 (83.3)
It was helpful to know the family caregiver who will be involved in providing care.	0 (0)	1 (16.7)	0 (0)	0 (0)	5 (83.3)
How helpful was it to have the patient identify the caregiver that he/she would like involved?	0 (0)	1 (16.7)	1 (16.7)	2 (33.3)	2 (33.3)
How helpful was it to have the patient indicate his/her communication preferences with the family caregiver who is involved in clinical care?	0 (0)	1 (16.7)	1 (16.7)	2 (33.3)	2 (33.3)
How helpful was it to allow the caregiver to report their information and support needs as a caregiver?	0 (0)	0 (0)	3 (50)	1 (16.6)	2 (33.3)
Overall how satisfied were you with the portal system to involve and support caregivers in patient care?	0 (0)	1 (16.7)	2 (33.3)	2 (33.3)	0 (0)
The caregiver was appropriately involved.	0 (0)	0 (0)	1 (16.7)	2 (33.3)	3 (50)
It allowed me to provide better care for the patient and his/her caregiver.	0 (0)	1 (16.7)	1 (16.7)	1 (16.7)	3 (50)
The method made patient appointments longer.	5 (83.3)	1 (16.7)	0 (0)	0 (0)	0 (0)

## Discussion

### Principal Results

This work demonstrates the usability of the patient-caregiver portal system among patients, caregivers, and clinicians in palliative care, and informs ongoing modifications prior to implementation among larger samples of patients and caregivers. Despite calls for engagement in care, caregivers remain inconsistently identified or asked about their needs [1,3,13,32]. Our patient-caregiver portal system is designed to integrate caregivers into care by recognizing patient autonomy, identifying caregivers needs, and connecting information to the care team. Caregiver engagement interventions such as ours have the potential to result in multitiered—caregiver, patient, and health system—benefit [14,17,33]. However, prior to broad implementation and assessment of such systems or strategies, a necessary first step is to explore stakeholder use, user experience, and perception of benefit or satisfaction. Given this work, we are now moving forward with broader implementation analysis on patient, caregiver, and system outcomes (ie, mental health, caregiving self-efficacy, quality of care, and unintended health service use).

Thus, this necessary, formative research sought to assess feasibility (of usage) and garner stakeholder feedback of our patient-caregiver portal system in the context of palliative oncology care. Our findings support effective patient and caregiver system use and perceived benefit. Specifically, all patients and nearly all caregivers answered their respective questions once they were logged into the system, and their responses were effectively transferred to the care team.

Our findings also suggest an ability to consistently identify information about patients and caregivers that has not otherwise been collected in a systematic manner. For example, the care team was informed about the communication preferences of patients, which most often involved shared communication with their caregiver. Similar to other findings [34,35], our findings show that there are instances in which the patient delegates communication. Without asking a patient’s preference, clinicians remain unaware of preferences in communication and could make incorrect assumptions about what the patient desires.

Similarly, this patient-caregiver portal system allowed the care team to receive information about caregivers, including their strain levels and information needs. Most of the caregivers in this sample felt prepared for their role and this might be due to the fact that they were further along in the care process and receiving palliative care. Despite feeling prepared, many caregivers expressed elevated caregiving-related strain, with nearly half reporting high emotional stress. This finding of elevated caregiving-related emotional stress replicates past findings specific to caregivers for persons with cancer [3]. Furthermore, a similar percentage indicated needing information about managing stress, while 36.8% (nearly 4 in 10) of caregivers requested information about managing financial strain. The downstream impact of financial toxicity on patients as well as caregivers is increasingly recognized as a gap to be addressed in the care process [36-38].

### Comparison With Prior Work

Alfano et al [13] have called for oncology care to become better equipped to recognize the needs of patients and caregivers in care. It is well-established that caregivers are often not asked about what they need to manage their own well-being as a caregiver, and these findings suggest unmet, and possibly, previously unrecognized needs. In assessments of US caregivers across varied caregiving contexts including oncology, caregivers have reported being rarely asked by health care providers about their needs [1,3]. According to the NAC’s 2016 report, “Cancer Caregiving in the U.S.,” slightly over half (54%) of caregivers for someone with cancer reported being asked by providers whether they needed information to care for the patient, while even fewer (29%) reported being asked if they needed information to care for themselves [3]. More recently, in the NAC and the American Association of Retired Persons report, “Caregiving in the U.S. 2020,” fewer (30%) caregivers indicated that the patient or care recipient’s provider had asked them about their needs to care for the patient, and less (13%) indicated being asked about their own self-care needs [1]. Given the findings, this system offers a feasible, and replicable, option to better integrate caregivers, recognize their needs, and provide appropriate resources, while also integrating information with the care team.

Overall, the user experience interviews from patients and caregivers and the clinician feedback survey suggest good to strong satisfaction with the system. Reasons for being satisfied among both patients and caregivers included ease of use and perceived value in including caregivers, particularly for emergencies or as cancer progresses. There was also notation of wanting to be able to indicate a specific or preferred caregiver so that the information was clear for the care team. Despite most patients and caregivers being satisfied with the system, it was also evident through user experience interviews that there were aspects that could be improved. Suggestions included having the system be more interactive and offer tailored information and potentially by enabling the care team to contact the caregiver by chat or email. Stakeholders also suggested initiating the system earlier in the care trajectory. Some evidence indicates that the early stages of caregiving can be most challenging due to a lack of preparation or information. For example, in studies with caregivers of patients diagnosed with head and neck cancer, information and caregiving skill-related needs were reported to be highest earlier in the care trajectory, including at diagnosis and during early treatment, while caregivers’ own psychological health-related needs were high throughout care [39].

Similarly, clinician feedback was both positive and constructive for areas of improvement. Specifically, moving forward, the system will continue to evolve to ensure collaboration at the cancer center and externally so that there are adequate resources to meet caregiver needs in particular. Feedback indicated that clinicians supported the system, particularly with respect to knowing about and supporting the caregiver. However, more resources will be required for this system to be expanded to a larger patient population. It might also require integration into the electronic medical record, increased support from the Social Work Department, and collaboration with existing community partners and nonprofits. Similar recommendations have been reported previously [24]. As the system evolves it is important to continue to explore issues of privacy with patient and caregiver information when portal information is shared, even among a patient and caregiver [40].

### Limitations

Despite the benefits of this pilot study, there are several limitations and notations for next steps. First, the sample of system users was predominantly non-Hispanic White. Future work should include a larger sample of patients and caregivers to allow for further exploration of differential use by broad sociodemographic factors, including age, race, ethnicity, and socioeconomic status. Our early phases of this developmental research did have greater racial and ethnic diversity, but it was also a small sample size [25]. Furthermore, though the focus on palliative care was intentional as a space that often integrates caregivers into care, it also represents a sample of patients who might have more advanced cancer or high symptom demands and thus impacts recruitment and retention. The goal of this study was to assess feasibility (ie, usability) of the patient-caregiver portal system among patients, caregivers, and clinicians to lend itself to next exploring the system among varied patient populations, including initiating such processes at patient diagnosis of cancer and outside of palliative care. Furthermore, as the primary objective of this study was to assess feasibility (usability and user experience), which was demonstrated to be high. The user experience interviews specifically allowed for comment on factors that might have enabled or limited an individual’s perception of use. However, we acknowledge that the impact of various human factors was not the focus of this particular study, and we have included this in the limitations section.

### Conclusions

The engagement of family caregivers in oncology is not universal or systematic. Our patient-caregiver portal system was developed to establish a systematic process that engages caregivers in care using an existing patient portal system. Our findings demonstrate system usability, including a systematic, and replicable way to identify caregiver needs and share with the care team in a way that is acceptable to both patients and caregivers, and perceived by clinicians to benefit clinical care.

## Abbreviations

AIR: American Institutes for Research
HIPAA: Health Insurance Portability and Accountability Act
NAC: National Alliance for Caregiving
RA: research assistant

## References

[1] (2020). Caregiving in the U.S.: 2020 report. National Alliance for Caregiving & AARP.

[2] (2015). Caregiving in the U.S.: 2015 report. National Alliance for Caregiving & AARP.

[3] Hunt GG, Longacre ML, Kent EE (2016). Cancer caregiving in the U.S.: an intense, episodic, and challenging care experience. National Alliance for Caregiving.

[4] Longacre ML, Brewer B, Hubbard A, Ashare RL, Patterson F (2021). Caregiver health by context: moderating effects of mental health and health behaviors. West J Nurs Res.

[5] Litzelman K, Yabroff KR (2015). How are spousal depressed mood, distress, and quality of life associated with risk of depressed mood in cancer survivors? Longitudinal findings from a national sample. Cancer Epidemiol Biomarkers Prev.

[6] Milbury K, Badr H, Fossella F, Pisters KM, Carmack CL (2013). Longitudinal associations between caregiver burden and patient and spouse distress in couples coping with lung cancer. Support Care Cancer.

[7] Longacre ML, Ridge JA, Burtness BA, Galloway TJ, Fang CY (2012). Psychological functioning of caregivers for head and neck cancer patients. Oral Oncol.

[8] Frambes D, Given B, Lehto R, Sikorskii A, Wyatt G (2018). Informal caregivers of cancer patients: review of interventions, care activities, and outcomes. West J Nurs Res.

[9] Ferrell B, Wittenberg E (2017). A review of family caregiving intervention trials in oncology. CA Cancer J Clin.

[10] Kent EE, Rowland JH, Northouse L, Litzelman K, Chou WYS, Shelburne N, Timura C, O'Mara A, Huss K (2016). Caring for caregivers and patients: research and clinical priorities for informal cancer caregiving. Cancer.

[11] Institute of Medicine, Committee on Quality of Health Care in America (2001). Crossing the Quality Chasm: A New Health System for the 21st Century.

[12] Committee on the Future Health Care Workforce for Older Americans, Board on Health Care Services, Institute of Medicine (2008). Retooling for an Aging America: Building the health care workforce.

[13] Alfano CM, Leach CR, Smith TG, Miller KD, Alcaraz KI, Cannady RS, Wender RC, Brawley OW (2019). Equitably improving outcomes for cancer survivors and supporting caregivers: a blueprint for care delivery, research, education, and policy. CA Cancer J Clin.

[14] Carman KL, Dardess P, Maurer ME, Workman T, Ganachari D, Pathak-Sen E (2014). A roadmap for patient and family engagement in Healthcare Practice and Research. The American Institutes for Research.

[15] Wolff JL, Feder J, Schulz R (2016). Supporting family caregivers of older Americans. N Engl J Med.

[16] Coleman EA (2016). Family caregivers as partners in care transitions: the caregiver advise record and enable act. J Hosp Med.

[17] Frampton SB, Guastello S, Hoy L, Naylor M, Sheridan S, Johnston-Fleece M (2017). Harnessing evidence and experience to change culture: a guiding framework for patient and family engaged care. NAM Perspectives.

[18] Clayman ML, Roter D, Wissow LS, Bandeen-Roche K (2005). Autonomy-related behaviors of patient companions and their effect on decision-making activity in geriatric primary care visits. Soc Sci Med.

[19] Wolff JL, Roter DL (2012). Older adults' mental health function and patient-centered care: does the presence of a family companion help or hinder communication?. J Gen Intern Med.

[20] Leighton C, Fields B, Rodakowski JL, Feiler C, Hawk M, Bellon JE, James AE (2020). A multisite case study of caregiver advise, record, enable act implementation. Gerontologist.

[21] (2021). Health Information National Trends Survey briefs number 45. National Cancer Institute.

[22] Johnson C, Richwine C, Patel V (2021). Individuals' access and use of patient portals and smartphone health apps, 2020. ONC Data Brief, no. 57.

[23] Gleason KT, Peereboom D, Wec A, Wolff JL (2022). Patient portals to support care partner engagement in adolescent and adult populations: a scoping review. JAMA Netw Open.

[24] Longacre ML, Chwistek M, Collins M, Odelberg M, Siemon MM, Keleher C, Fang C (2021). Palliative care clinicians' perspectives of an integrated caregiver patient-portal system in oncology. Cancer Care Res Online.

[25] Longacre ML, Keleher C, Chwistek M, Odelberg M, Siemon M, Collins M, Fang CY (2021). Developing an integrated caregiver patient-portal system. Healthcare (Basel).

[26] Wolff J, Guan Y, Boyd CM, Vick J, Amjad H, Roth DL, Gitlin LN, Roter DL (2017). Examining the context and helpfulness of family companion contributions to older adults' primary care visits. Patient Educ Couns.

[27] Wolff JL, Clayman ML, Rabins P, Cook MA, Roter DL (2015). An exploration of patient and family engagement in routine primary care visits. Health Expect.

[28] Longacre ML, Miller MF, Fang CY (2023). The psychometric properties of a caregiving-related strain scale in oncology. Qual Life Res.

[29] Tanco K, Park JC, Cerana A, Sisson A, Sobti N, Bruera E (2017). A systematic review of instruments assessing dimensions of distress among caregivers of adult and pediatric cancer patients. Palliat Support Care.

[30] Dalrymple PW, Rogers M, Zach L, Luberti A (2018). Understanding internet access and use to facilitate patient portal adoption. Health Informatics J.

[31] Bradley EH, Curry LA, Devers KJ (2007). Qualitative data analysis for health services research: developing taxonomy, themes, and theory. Health Serv Res.

[32] Nightingale CL, Sterba KR, McLouth LE, Kent EE, Dressler EV, Dest A, Snavely AC, Adonizio CS, Wojtowicz M, Neuman HB, Kazak AE, Carlos RC, Hudson MF, Unger JM, Kamen CS, Weaver KE (2021). Caregiver engagement practices in National Cancer Institute Clinical Oncology Research Program settings: implications for research to advance the field. Cancer.

[33] Van Houtven CH, Voils CI, Weinberger M (2011). An organizing framework for informal caregiver interventions: detailing caregiving activities and caregiver and care recipient outcomes to optimize evaluation efforts. BMC Geriatr.

[34] Longacre ML, Miller MF, Golant M, Buzaglo JS, Zaleta AK (2018). Care and treatment decisions in cancer: the role of the family caregiver. J Oncol Navig Surviv.

[35] Schumacher FA, Helenowski IB, Oswald LB, Gonzalez BD, Benning JT, Morgans AK (2022). Treatment decision-making in metastatic prostate cancer: perceptions of locus of control among patient, caregiver, and physician triads. Patient Prefer Adherence.

[36] Goyal N, Day A, Epstein J, Goodman J, Graboyes E, Jalisi S, Kiess AP, Ku JA, Miller MC, Panwar A, Patel VA, Sacco A, Sandulache V, Williams AM, Deschler D, Farwell DG, Nathan C, Fakhry C, Agrawal N (2022). Head and neck cancer survivorship consensus statement from the American Head and Neck Society. Laryngoscope Investig Otolaryngol.

[37] Alzehr A, Hulme C, Spencer A, Morgan-Trimmer S (2022). The economic impact of cancer diagnosis to individuals and their families: a systematic review. Support Care Cancer.

[38] de Moor JS, Williams CP, Blinder VS (2022). Cancer-related care costs and employment disruption: recommendations to reduce patient economic burden as part of cancer care delivery. J Natl Cancer Inst Monogr.

[39] Wang T, Mazanec S, Voss J (2021). Needs of informal caregivers of patients with head and neck cancer: a systematic review. Oncol Nurs Forum.

[40] Applebaum AJ, Kent EE, Lichtenthal WG (2021). Documentation of caregivers as a standard of care. J Clin Oncol.

